# Optical Activation of the Dorsal Horn of the Thoracic Spinal Cord Prevents Ventricular Arrhythmias in Acute Myocardial Ischemia-Reperfusion Rats

**DOI:** 10.3389/fcvm.2022.753959

**Published:** 2022-02-07

**Authors:** Yong Wu, Zhongxu Luo, Zhengtao Hu, Kun Lv, Yinhua Liu, Deguo Wang

**Affiliations:** ^1^Department of Gerontology, First Affiliated Hospital of Wannan Medical College (Yijishan Hospital), Wuhu, China; ^2^Key Laboratory of Non-coding RNA Transformation Research of Anhui Higher Education Institution (Wannan Medical College), Wuhu, China; ^3^Department of Pathology, First Affiliated Hospital of Wannan Medical College (Yijishan Hospital), Wuhu, China

**Keywords:** ischemia-reperfusion, ventricular arrhythmia, optogenetic, spinal cord, sympathetic nerve activity

## Abstract

**Background and Objectives:**

Spinal cord stimulation can prevent myocardial ischemia and reperfusion arrhythmias, but the relevant neurons and mechanisms remain unknown. Thus, this study applied optogenetic techniques to selectively activate glutamatergic neurons at the thoracic spinal cord (T1 segment) for examining the anti-arrhythmia effects during acute myocardial ischemic-reperfusion.

**Methods:**

Adeno-associated viruses (AAVs) carrying channelrhodopsin-2 (ChR2, a blue-light sensitive ion channel) CaMKIIα-hChR2(H134R) or empty vector were injected into the dorsal horn of the T1 spinal cord. Four weeks later, optogenetic stimulation with a 473-nm blue laser was applied for 30 min. Then, the myocardial ischemia-reperfusion model was created by occlusion of the anterior descending coronary artery for ischemia (15 min) and reperfusion (30 min). Cardiac electrical activity and sympathetic nerve activity were assessed continuously.

**Results:**

CaMKIIα-hChR2 viral transfection is primarily expressed in glutamatergic neurons in the spinal cord. Selective optical stimulation of the T1 dorsal horn in the ChR2 rat reduced the ventricular arrhythmia and arrhythmia score during myocardial ischemia-reperfusion, preventing the over-activation of cardiac sympathetic nerve activity. Additionally, optical stimulation also reduced the action potential duration at the 90% level (APD90) and APD dispersion.

**Conclusion:**

Selective optical stimulation T1 glutamatergic neurons of dorsal horn prevent ischemia-reperfusion arrhythmias. The mechanism may be associated with inhibiting sympathetic nervous system overexcitation and increasing APD dispersion during myocardial ischemia-reperfusion.

## Introduction

Acute myocardial ischemia causes cardiac injury, arrhythmias and excessive sympathetic excitation through complex spinal cord circuits (1). Sympathetic hyperactivity is associated with arrhythmia (2) and neural remodeling (3). Previous studies have reported that epidural anesthesia or spinal cord stimulation (SCS) reduces sympathetic excitation and malignant ventricular arrhythmias (4). SCS modulates afferent nervous signals (5), sympathetic neurotransmitter release (6, 7), and autonomic dysregulation (4). Multiple interactions among neurons in the thoracic spinal cord may be involved in cardiac sympathetic overactivation (8).

However, the low specificity and permissive effects on the spinal cord hinder the illustration of the detailed mechanism of SCS. The optogenetic selective modulation of the spinal cord neurons allows for determining the role of neuronal circuits (9, 10). The selective expression of the photosensitive protein (channel rhodopsin-2, ChR2) in neurons makes light-induced neural electrical activity possible (11, 12). Importantly, a micro-wireless optogenetic device was designed for optogenetic stimulation spinal cord in freely moving rodents (13, 14). A study showed that the optogenetic stimulation of sympathetic preganglionic neurons in the spinal cord regulated cardiorespiratory activity (15).

Previous studies have shown that experimental coronary occlusion causes abnormal sympathetic reflex, myocardial non-uniform recovery of excitability, and dispersion of refractoriness in the myocardium (16–18). The dorsal root section reduces ischemia arrhythmias through the interruption of the sympathetic reflex (19). Our recent studies have also found that abolishing afferent nerve activities at the dorsal horn prevents arrhythmia and cardiac remodeling (2, 20). In this study, we try to determine whether optical activation of the glutamatergic neurons in the dorsal horn affects sympathetic reflex activity during acute myocardial ischemia-reperfusion.

## Methods

### Animals and Groups

Adult male rats (Sprague Dawley, weight 180–210 g) were randomized into the ChR2 group (*n* = 10) and control group (*n* = 10) who received adeno-associated virus (AAV) or empty control. All experimental procedures were approved by the Animal Care and Use Ethics Committee of Yijishan Hospital and performed in accordance with the Guideline for the Care and Use of Laboratory Animals of the National Institutes of Health's guiding principles.

### Virus Injection

**Figure 1:**
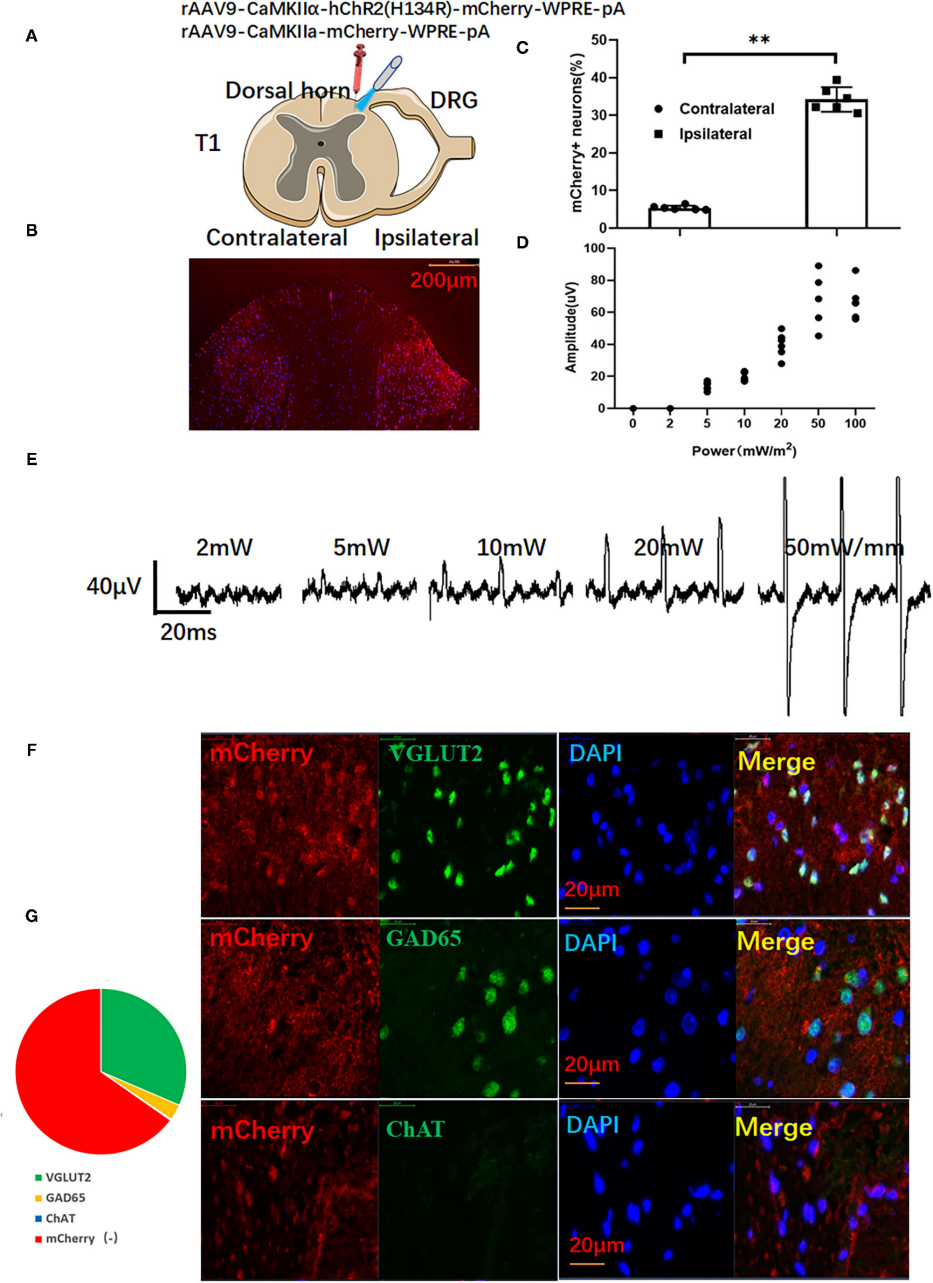
The activation of ChR2-expressing glutamatergic neurons in the dorsal horn induces myoelectric potentials of the left upper limb. **(A)** Adeno-associated virus (AAV)-carrying ChR2 or empty vector-transfected left dorsal horn (red syringe) for optical stimulation by a blue laser (473 nm). **(B)** Representative images of mCherry in the dorsal horn. **(C)** Viral transfection efficiency. **(D)** The optical stimulation of dorsal horn neurons induces electromyography (EMG) of the left upper limb and representative traces of EMG **(E)**. **(F)** mCherry-positive cells expressed major glutamatergic neuron marker VGLUT2, minor GABAergic neuron marker GAD65, but not cholinergic neuron marker ChAT. **(G)** The relative ratio of mCherry-positive neurons. A paired *t*-test was used to compare mCherry positive cells between ipsilateral and contralateral dorsal horns. (*n* = 10 for each group; **P < 0.01).

All rats were placed in a spinal stereotaxic frame with forceps clamped to spinal processes C2 and T2 after anesthetizing with 10% chloral hydrate (0.3 g/kg body weight, i.p.). Spinal surgery was performed as previously described (21). AAV9-containing Ca(2+)/calmodulin-activated protein kinase II-α (CaMKIIα) prompter with or without -hChR2(H134R) (PackGene Biotech, 10^13^ vg/ml) was drawn up into a 10 μl Hamilton syringe with a glass pipette and pulled to a 20 μm tip (Hamilton, Shanghai, Ch). Then AAVs (3 μl) were injected into the dorsal horn (0.5 mm depth) under control by a stereotaxic micromanipulator through previously reported methods (11). The injection and needle retention were kept for 10 min to let the virus penetrate evenly into the dorsal horn (Figure 1A).

### Electromyography and Optical Stimulation

Needle electrodes were inserted into the left upper limb 4 weeks after virus injection to record electromyography (EMG) (100–30,000 Hz filters) offline (RM6240, Chengdu, China). After EMG implantation, the muscles were carefully dissected from C2 to T2. The animals were then clamped to the dorsal C2 and T1 processes of the spinal cord in the spinal stereotactic frame to prevent spinal cord movement during the whole process of optical stimulation (11) (Details were shown in Supplementary Materials). Optical stimulation was performed by a laser stimulator with blue light (473 nm, Newton Co., Ltd., Hangzhou, China) through a 200 μm diameter optical fiber. Before ischemia and reperfusion, the rats received optogenetic activation for 30 min (1 min laser on and 4 min laser off, repeated 6 times). Different stimulation parameters (0, 2, 5, 10, 20, 50, and 50 mW, 10 ms pulse at frequency of 1 Hz) were applied to each side of the dorsal horn. As shown in Figures 1D,E, EMG gradually increased with enhanced stimulation intensity. Stimulation parameters with a pulse width of 10 ms and intensity of 20 mW/m^2^ were selected as the final study. According to previous reports, SCS with 90% of the motor threshold can protect the ischemic heart (22). In this study, we selected proper optical stimulation parameters which induce EMG but no limb movement (11).

### Ischemia-Reperfusion Model

According to our previous report, we created a rat model of myocardial ischemia-reperfusion (23). In brief, rats were subjected to occlusion of the coronary artery to induce myocardial ischemia for 15 min and then released for 30 min for reperfusion. Successful myocardial ischemia was judged by the ST-segment elevation on ECG and pallor changes in the heart immediately after ligation. Blue light stimulation was administered 30 min prior to ischemia.

### Cardiac Monophasic Action Potential Measurement and Analysis

Monophasic action potentials (MAPs) were recorded and analyzed using a Biosignal analysis software according to our previously developed method (RM6240, Chengdu, China) (24). In brief, a pair of electrodes were placed on the base and free wall of the heart, and the control electrode was connected to the skin. The MAP data were recorded and stored in a computer for further analysis (24, 25) (**Figure 3A**). APD90 was defined as the duration of 90% repolarization (APD90). APD dispersion (APDd) was calculated by subtracting the minimum APD from the maximum APD.

### Arrhythmia Analysis

Electrocardiography (ECG) recording and arrhythmia identification complied with Lambeth Convention criteria (26). Ischemia-reperfusion induced arrhythmia analysis was used to calculate the duration of ventricular arrhythmia (VA), ventricular fibrillation (VF), and ventricular arrhythmia score, shown as follows: 0: < 10 ventricular premature contractions (VPCs); 1: ≥ 10 VPCs; 2: 1–5 episodes of ventricular tachycardia (VT); 3: > 5 VTs or 1 ventricular fibrillation (VF), 4: 2–5 episodes of VF; 5: > 5 episodes of VF; 6: VT, VF, or both for a duration ≤ 300 s; 7: VT, VF, or both for a duration > 300 s.

### Measurement of Cardiac Sympathetic Nervous Activity

Cervical sympathetic nerve activity (CSNA) was recorded as previously described (2, 20). In brief, CSNA was recorded by a biological data acquisition system (RM6240, Chengdu, China) using two pairs of hook electrodes (2 mm interval) for offline further analysis. The CSNA amplitude was continuously recorded during baseline, light stimulation, myocardial ischemia, and reperfusion phases.

### Immunohistochemistry

The fluorescent protein mCherry, the glutamatergic neuron marker VGLUT2, the GABAergic neuron marker GAD65, and the cholinergic neuron marker ChAT (VGLUT2, 1:200; GAD65, 1:300; ChAT, 1:100; Abcam) were used for colocalization analysis of the ratio of different neurons. Myocardial tissue sections were processed for immunofluorescence as before (24).

### Statistical Analysis

All data were expressed as means ± SD. Student *t*-test was used to evaluate the difference between the ChR2 and Control groups. A paired *t*-test was used to compare variables with or without optical stimulation. Significant differences among baseline, ischemia, and reperfusion were evaluated using one-way ANOVA, followed by a *post-hoc* Newman–Keuls multiple comparison test. Statistical analyses were performed using SPSS 16.0 (IBM, New York, USA), and *P* < 0.05 was considered significant.

## Results

### Activation of ChR2-Expressing Dorsal Horn Neurons Induces Left Upper Limb Muscle Activity and Myoelectric Potentials in Anesthetized Rats

As shown in Figure 1B, red signals of mCherry can be seen at the ipsilateral dorsal horn. However, there were only a few mCherry positive signals in the contralateral dorsal horn (Figures 1B,C). As shown in Figures 1D,E, an increased EMG was recorded at the left upper limb of rats. When the stimulation intensity was above 50 mW/mm, forelimb contraction could be observed in rats. Thus, stimulation parameters (20.3 ± 2.8 mW/mm, pulse width: 10 ms) were selected as subsequent spinal cord stimulation. As shown in Figures 1D,F, immunofluorescence double staining showed that most merged neurons were co-stained with the glutamatergic neuron marker VGLUT2 and mCherry(+). Only a few neurons were co-stained with GABAergic neuron marker GAD65. Almost no neurons co-expressed the cholinergic neuron marker ChAT with mCherry (Figure 1G).

### Selective Activation of Glutamatergic Neuron in Dorsal Horn Prevents Ischemia-Reperfusion Arrhythmia

**Figure 2:**
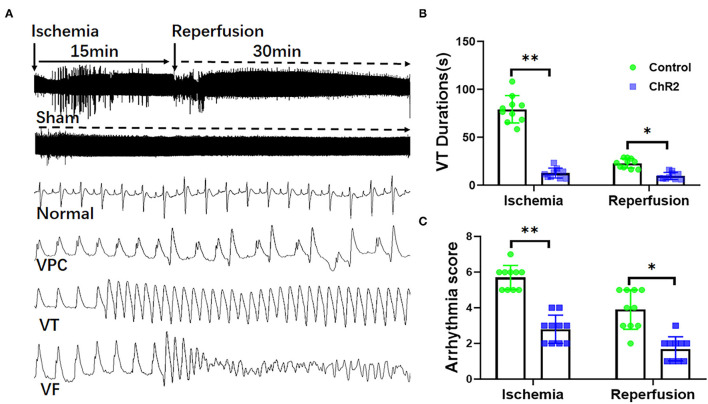
Selective optical stimulation of the dorsal horn prevents arrhythmias during myocardial ischemia-reperfusion. Representative traces of ECG and arrhythmias including ventricular premature contraction (VPCs), ventricular tachycardias (VTs), and ventricular fibrillation (VF) during 15 min of ischemia and 30 min of reperfusion **(A)**. Analysis of VT and VF showed that optical stimulation of the dorsal horn significantly inhibited VT duration **(B)** and arrhythmia score **(C)**. Data are expressed as mean ± SD. A paired *t*-test was used to compare parameters before and after light stimulation and a *t*-test was used to compare differences between the ChR2 and control groups (*n* = 10 for each group; *P < 0.05, **P < 0.01).

As shown in Figure 2A, both myocardial ischemia and reperfusion-induced ventricular arrhythmias like VPCs, VT, and VF. Optical stimulation significantly reduced the VT durations during ischemia (12.6 ± 5.2 s vs. 79.2 ± 14.3 s, *P* < 0.05) and reperfusion (9.6 ± 3.6 s vs. 22.8 ± 4.9 s, *P* < 0.05; Figure 2B). Optical stimulation significantly reduces the arrhythmia score of VA during the ischemia (2.8 ± 0.8 vs. 5.7 ± 0.7, *P* < 0.01) and reperfusion (1.7 ± 0.7 vs. 3.9 ± 1.1, *P* < 0.05; Figure 2C).

### Selective Activation of Glutamatergic Neuron in Dorsal Horn Prevents Prolongation of APD in Ischemia-Reperfusion Heart

**Figure 3:**
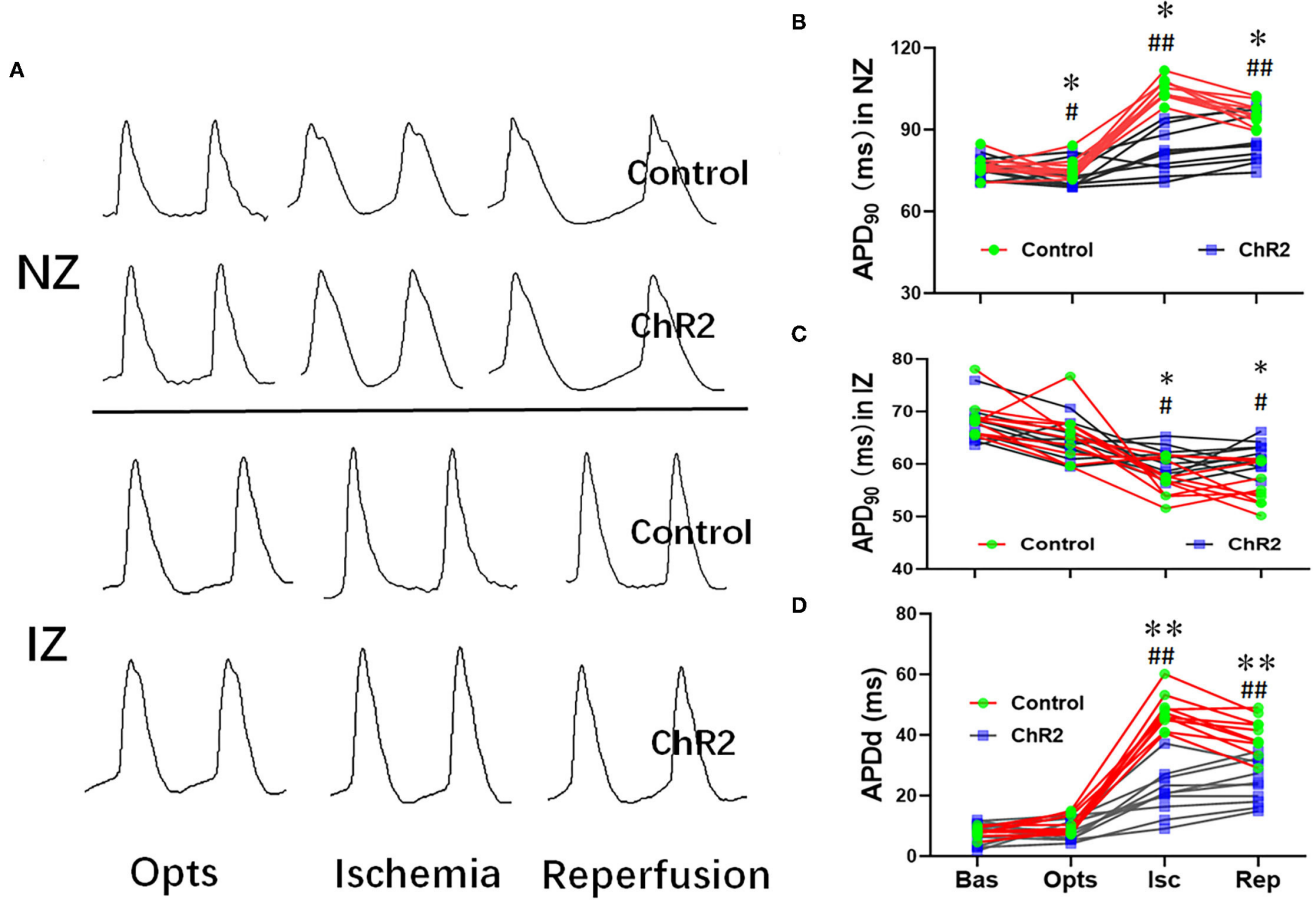
Selective optical stimulation of the dorsal horn prevents cardiac APD abnormalities during myocardial ischemia-reperfusion. Representative traces of monophasic action potentials (MAPs) both none-infarcted zone (NZ) and infarcted zone (IZ) during 15 min of ischemia and 30 min of reperfusion **(A)**. Quantitative analysis of APD_90_ at baseline (Bas), optical stimulation (Opts), ischemia (Isc), and reperfusion (Rep) from NZ **(B)** and IZ **(C)**. **(D)** Optical stimulation of ChR2 can significantly decrease APD dispersion during myocardial ischemia-reperfusion. Data are expressed as mean ± SD. A paired *t*-test was used to compare parameters before and after light stimulation and a *t*-test was used to compare differences between the ChR2 and control groups (*n* = 10 per group; *P < 0.05, **P < 0.01 vs. Control; ^#^*P* < 0.05, ^##^*P* < 0.01 vs. Baseline).

During the myocardial ischemia and reperfusion, APD was significantly prolonged in the non-infarct zone (NZ) but shortened in the infarct zone (NZ) (Figures 3A–C). As a result, APD dispersion in the control group increased significantly during myocardial ischemia (47.7 ± 5.7 ms vs. 7.8 ± 1.5 ms, *P* < 0.01) and reperfusion (39.9 ± 6.2 ms vs. 7.8 ± 1.5 ms, *P* < 0.01). Compared with the control group, optical stimulation significantly reduced APD dispersion both in ischemia (21.2 ± 7.9 ms vs. 47.7 ± 5.7 ms, *P* < 0.01) and reperfusion phrase (24.2 ± 7.0 vs. 39.9 ± 6.2 ms, *P* < 0.01; Figure 3D).

### Selective Activation of the Dorsal Horn Prevents Ischemia-Reperfusion Cardiac Sympathetic Hyperactivity

**Figure 4:**
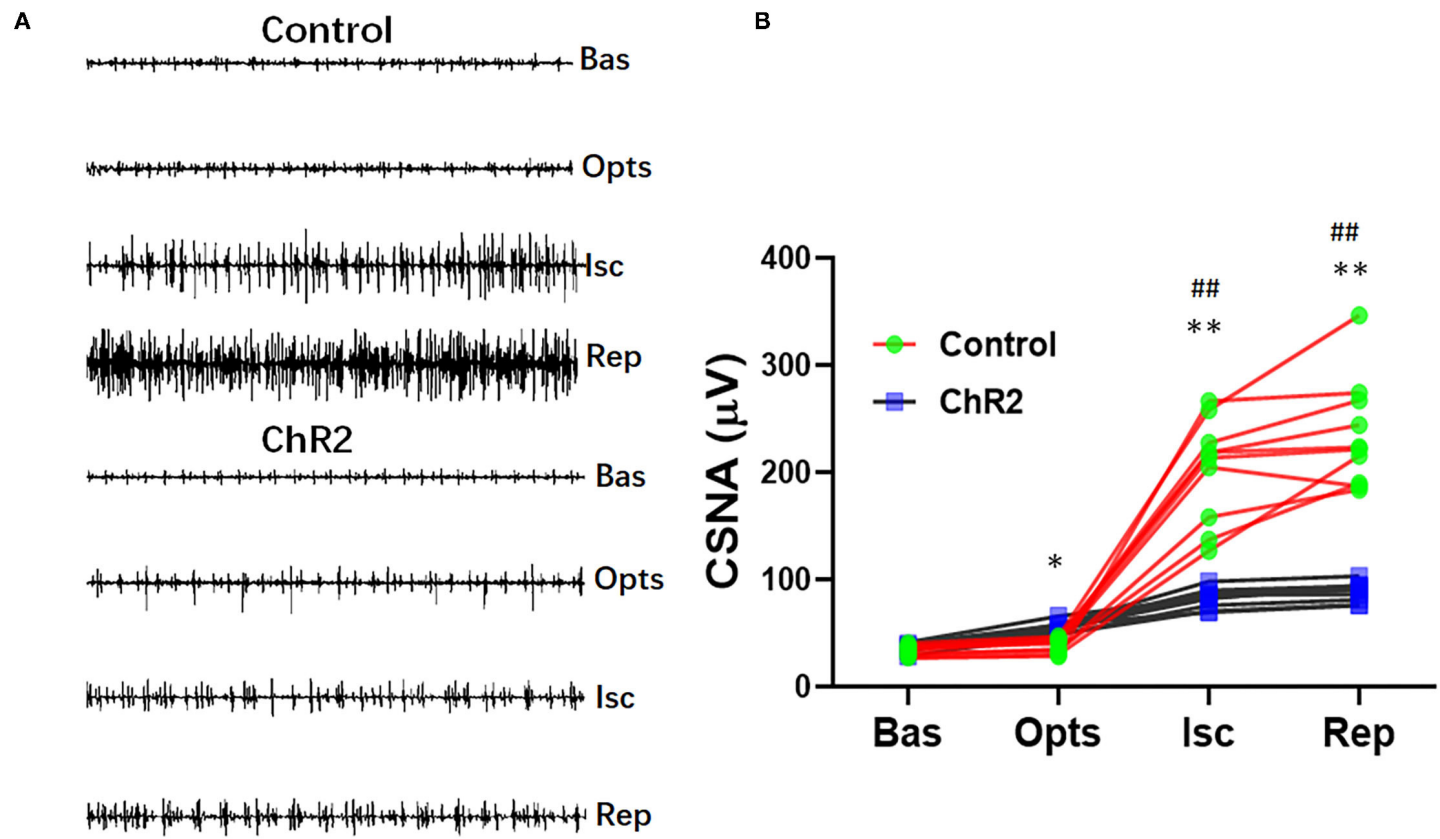
Selective optical stimulation of the dorsal horn prevents cardiac sympathetic hyperactivity during myocardial ischemia-reperfusion. **(A)** Representative traces of cardiac sympathetic nerve activity (CSNA) during 15 min of ischemia and 30 min of reperfusion from control and ChR2 rats. **(B)** Quantitative analysis of CSNA at baseline (Bas), optical stimulation (Opts), ischemia (Isc), and reperfusion (Rep). Data are expressed as mean ± SD. A paired *t*-test was used to compare parameters before and after optical stimulation and a *t*-test was used to compare differences between the ChR2 and control groups (*n* = 10 per group; *P < 0.05, **P < 0.01 vs. Control; ^##^*P* < 0.01 vs. Baseline).

Optical stimulation of the dorsal horn neurons significantly increased CSNA compared with the control group (55.3 ± 4.9 vs. 38.9 ± 6.6 μV, *P* < 0.05). CSNA significantly increased during myocardial ischemia and reperfusion in the control group (202.9 ± 47.7 vs. 33.6 ± 4.7 μV, *P* < 0.01; Figures 4A,B). However, optical stimulation of dorsal horn glutamatergic neurons significantly prevented CSNA during ischemia (82.6 ± 8.7 vs. 202.9 ± 47.7 μV, *P* < 0.01) and reperfusion (88.7 ± 8.7 vs. 235.3 ± 50.2 μV, *P* < 0.01; Figure 4B).

## Discussion

Optogenetic methods have been used to activate or inhibit specific neurons by stimulating selectively the ChR2 or Arc in target cells (10). It is reported that different promoters can selectively express a photosensitive protein to optically stimulate specific neurons in the spinal cord (11). Micro-wireless LED-optic devices can also optogenetic stimulate the spinal cord in freely moving rodents (13, 14). Previous studies have shown that optical stimulation dorsal horn causes muscle movement in animals (11, 12). As a preliminary study, we demonstrated that selectively activating glutamatergic neurons of the dorsal horn in T1 can prevent ventricular arrhythmias during myocardial ischemia and reperfusion and reduce sympathetic discharges and APD dispersion.

Previous studies have shown that SCS significantly ameliorates myocardial ischemia and ventricular arrhythmias during ischemia and reperfusion (4, 22). SCS significantly inhibits the abnormal activity of neurons in the dorsal horn at the T1–4 level (5). In this study, we confirmed that the pre-activation of glutamatergic neurons in the T1 dorsal horn inhibited ventricular arrhythmias during ischemia-reperfusion, suggesting that glutamatergic neurons played an important role in the protective mechanisms of SCS. However, further studies are still needed to clarify the interplay among spinal neurons.

Abnormal cardiac APD or dispersion of refractoriness is an important mechanism of ischemia-reperfusion arrhythmias (16, 18, 27). SCS alleviates the shortness of repolarization and dispersion of repolarization in ischemic heart SCS (4, 28). In this study, during ischemia and reperfusion, the activation of glutamatergic neurons in the dorsal horn can shorten the APD in the none ischemic area and prolong the APD in the ischemic area, so as to improve the APD dispersion of the heart.

This study indicates that glutamatergic neurons in the dorsal horn may be involved in the protective mechanism of SCS. A study showed that myocardial ischemia led to partial spinal cord neuron activity, including dorsal horn neurons and interneurons (8). SCS decreases myocardial injury by producing protective substances such as dynorphin in the local spinal cord (6, 29). Previous studies have confirmed the possibility of optical stimulation of specific neurons of the spinal cord to illustrate spinal functional circuits (11, 15, 30–32). This study found that activation of glutamatergic neurons prevented cardiac sympathetic nerve discharge during ischemia-reperfusion. These results suggest that the pre-activation of glutamatergic neurons in the dorsal horn inhibits followed overexcitation of cardiac sympathetic during myocardial ischemia-reperfusion.

As a preliminary study, this work has some limitations. First, ChR2 was also expressed in some GABAergic neurons. We are not sure whether GABAergic neurons participate in the anti-arrhythmic effect in this study. Second, further study is required to determine the long-term effect of the optical stimulation of glutamatergic neurons in the dorsal horn. Third, this study did not determine whether optical stimulation of the dorsal horn terminates arrhythmias during myocardial ischemia and reperfusion.

## Conclusion

The present study showed that specific stimulation of glutamatergic neurons in the dorsal horn of the T1 spinal cord can significantly inhibit arrhythmias and improve APD abnormalities during ischemia-reperfusion. The protective mechanism may be associated with the regulation of the cardiac-spinal reflex circuit and the inhibition of overexcitation of the cardiac sympathetic nerve during the ischemia-reperfusion.

## Data Availability Statement

The original contributions presented in the study are included in the article/Supplementary Material, further inquiries can be directed to the corresponding author/s.

## Ethics Statement

The animal study was reviewed and approved by Yijishan Hospital Institutional Animal Care and Use Committee.

## Author Contributions

DW and KL: participated in research design. YW, ZL, YL, and ZH: conducted experiments. DW and YW: performed data analysis. DW: wrote or contributed to the writing of the manuscript. All authors contributed to the article and approved the submitted version.

## Funding

This work was supported by grants from the National Natural Science Foundation of China (Nos. 81670301 to DW).

## Conflict of Interest

The authors declare that the research was conducted in the absence of any commercial or financial relationships that could be construed as a potential conflict of interest.

## Publisher's Note

All claims expressed in this article are solely those of the authors and do not necessarily represent those of their affiliated organizations, or those of the publisher, the editors and the reviewers. Any product that may be evaluated in this article, or claim that may be made by its manufacturer, is not guaranteed or endorsed by the publisher.
