# Supplementary material for: Optical Activation of the Dorsal Horn of the Thoracic Spinal Cord Prevents Ventricular Arrhythmias in Acute Myocardial Ischemia-Reperfusion Rats
Source: Front Cardiovasc Med. 2022 Feb 7;9:753959. doi: 10.3389/fcvm.2022.753959 (PMC8858961; doi:10.3389/fcvm.2022.753959)

**MATERIALS AND METHODS**

**1.1 Animals and groups**

Adult male rats (Sprague Dawley, weight 180–210g) were randomized into the ChR2 group (n=10) and Control group (n=10) whose received AAV virus and empty control. All experimental procedures were approved by Animal Care and Use Ethics Committee of Yijishan Hospital and performed in accordance with Guideline for the Care and Use of Laboratory Animals of the National Institutes of Health’s guiding principles.

**1.2 Virus injection**

All rats were placed in a spinal stereotaxic frame with forceps clamped to spinal processes C2 and T2 after anesthetizing by 10% chloral hydrate (0.3g/kg body weight, i.p.). Then, lidocaine (1 mg/kg) and enrofloxacin (5mg/kg) injected intramuscularly for reducing pain and infection. An incision was made in the skin and muscle layers over spinal segments C2-T2([Mondello et al., 2021](#_ENREF_2)). The surgery details were performed as previous reports([Keefe et al., 2019](#_ENREF_1)). AAV virus containing rAAV9-CaMKIIa-mCherry-WPRE-pA or rAAV9- CaMKIIα- hChR2 (H134R) -mCherry-WPRE-pA (PackGene Biotech, 10^13^ vg/ml) was drawn up into a10μl Hamilton syringe (Hamilton, Shanghai, Ch) with a glass pipette pulled to a 20μm tip. Then AAVs (3ul) were injected into the dorsal horn (0.5 mm depth) under controlling by stereotaxic micromanipulator as previous reported methods([Mondello et al., 2018](#_ENREF_3)). The injection and needle retention were kept for 10 minutes to allow the virus was evenly infiltrated into the dorsal horn of spinal cord. After AAV injections, Gelfoam was positioned over the exposed spinal cord and muscles and skin were sutured in layers ([Mondello et al., 2018](#_ENREF_3)).

**1.3 Electromyography (EMG)**

A pair of needle electrodes were insert into biceps of left forelimb([Mondello et al., 2018](#_ENREF_3)). A single pulse stimulus was given to test the position of electrodes in the target muscle. After covering tissue glue, the electrode wires were connected to a Multiple-channel Biosignal Record and Analysis system (RM6240, Chengdu, China) for recording optically evoked EMG activity. EMG data were recorded with sampling frequency of 20kHz, scanning speed of 40ms/div, and filter frequency from 100–30000 Hz. After optically stimulation, a significant muscle electrical response above noise was recorded as shown in figure 1E.

**1.4 Optical stimulating procedure.**

Optical stimulating was performed 4 weeks after AAV injection. Rats were anesthetized with 10% chloral hydrate (0.3g/kg body weight, i.p.). Following EMG implantation, an incision was made from C2 to T2 through dissection muscle carefully. Then clamped the animal at dorsal spinal processes C2 and T1 in spinal stereotaxic frame for prevent spinal moving throughout optical stimulation procedure([Mondello et al., 2018](#_ENREF_3)). Optical stimulation of spinal cord was performed by a laser stimulator with blue light (473 nm, Newton Co., Ltd, Hangzhou, China) through a 200μm diameter optical fiber. Optical stimulations with different parameters (0,2 5,10,20,50 and 50mW, 10ms pulse at frequency of 1Hz) were applied to each side of dorsal horn. As shown in Figure 1D and E, EMG gradually increased with enhanced stimulation intensity. Stimulation parameter with a pulse width of 10ms and intensity of 20 mW/m^2^ were selected as final study. According to previous reports that SCS with 90% of the motor threshold can protect ischemic heart ([Odenstedt et al., 2011](#_ENREF_4)), we selected the optical stimulation parameters as in which inducing EMG but no limb movement([Mondello et al., 2018](#_ENREF_3)). Before ischemia and reperfusion, rats receive 30min optogenetic activation (1min laser on and 4min laser-off, repeated for 6 times).

**1.5 Ischemia-reperfusion model**

Myocardial ischemia-reperfusion model was made in rat according to our previous study ([Sun et al., 2014](#_ENREF_5)). In brief, rats were subjected to coronary artery occlusion to induce myocardial ischemia for 15min and artery reopening for 30min for reperfusion. The successful occlusion was judged by the ST-segment elevation on ECG and pallor transition in heart immediately after ligation.

**1.6 Cardiac monophasic action potential measurement and analysis**

Monophasic action potentials (MAPs) were recorded and analyzed according to our previously developed method using a Biosignal analysis software (RM6240, Chengdu, China) ([Wang et al., 2016](#_ENREF_7)). To record monophasic action potentials (MAP), a pair of electrodes were placed on the base and free wall of the heart, and the control electrode was connected to the skin. The MAP data were recorded and stored in computer for further analysis ([Wang et al., 2011](#_ENREF_6);[Wang et al., 2016](#_ENREF_7))(Fig 3A). APD90 was defined as duration of 90% repolarization (APD90). APD dispersion (APDd) was calculated by subtracting the minimum APD from the maximum APD.

**References**

Keefe, K.M., Junker, I.P., Sheikh, I.S., Campion, T.J., and Smith, G.M. (2019). Direct Injection of a Lentiviral Vector Highlights Multiple Motor Pathways in the Rat Spinal Cord. *J Vis Exp*.

Mondello, S.E., Pedigo, B.D., Sunshine, M.D., Fischedick, A.E., Horner, P.J., and Moritz, C.T. (2021). A micro-LED implant and technique for optogenetic stimulation of the rat spinal cord. *Exp Neurol* 335**,** 113480.

Mondello, S.E., Sunshine, M.D., Fischedick, A.E., Dreyer, S.J., Horwitz, G.D., Anikeeva, P., Horner, P.J., and Moritz, C.T. (2018). Optogenetic surface stimulation of the rat cervical spinal cord. *J Neurophysiol* 120**,** 795-811.

Odenstedt, J., Linderoth, B., Bergfeldt, L., Ekre, O., Grip, L., Mannheimer, C., and Andrell, P. (2011). Spinal cord stimulation effects on myocardial ischemia, infarct size, ventricular arrhythmia, and noninvasive electrophysiology in a porcine ischemia-reperfusion model. *Heart Rhythm* 8**,** 892-898.

Sun, X., Zhong, J., Wang, D., Xu, J., Su, H., An, C., Zhu, H., and Yan, J. (2014). Increasing glutamate promotes ischemia-reperfusion-induced ventricular arrhythmias in rats in vivo. *Pharmacology* 93**,** 4-9.

Wang, D., Zhang, F., Shen, W., Chen, M., Yang, B., Zhang, Y., and Cao, K. (2011). Mesenchymal stem cell injection ameliorates the inducibility of ventricular arrhythmias after myocardial infarction in rats. *Int J Cardiol* 152**,** 314-320.

Wang, D., Zhu, H., Yang, Q., and Sun, Y. (2016). Effects of relaxin on cardiac fibrosis, apoptosis, and tachyarrhythmia in rats with myocardial infarction. *Biomed Pharmacother* 84**,** 348-355.

**FS1: Ventricular arrhythmias during myocardial ischemia and reperfusion.** Reperfusion arrhythmia like ventricular premature contraction (VPCs), ventricular tachycardias (VTs) and ventricular fibrillations (VFs) appeared majorly at the early stage (5 min) during the 30 minutes of reperfusion.


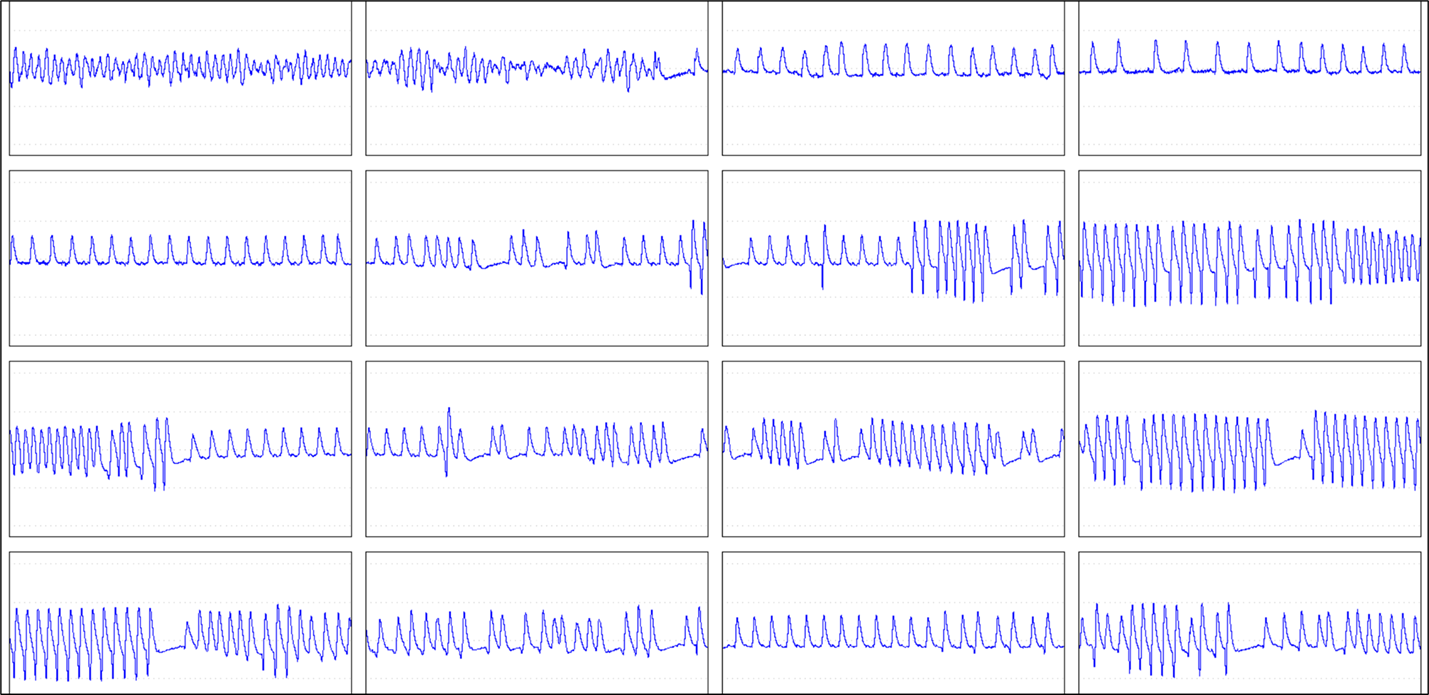

Supplement: Supplementary file 6 [file Table_6.DOCX]
